# Template-Assisted Electrodeposited Copper Nanostructres for Selective Detection of Hydrogen Peroxide

**DOI:** 10.3390/molecules29194571

**Published:** 2024-09-26

**Authors:** Bommireddy Naveen, Sang-Wha Lee

**Affiliations:** Department of Chemical and Biological Engineering, Gachon University, 1342 Seongnam-daero, Seongnam-si 13120, Gyeonggi-do, Republic of Korea; naveenb@gachon.ac.kr

**Keywords:** electrodeposition, hydrogen peroxide, surfactants, chronoamperometry

## Abstract

In this study, we demonstrate the electrodeposition of copper nanoparticles (NPs) on pencil graphite electrodes (PGEs) utilizing sodium dodecyl sulphate (SDS) as a soft template. The utilization of the surfactant had an impact on both the physical arrangement and electrochemical characteristics of the modified electrodes. The prepared Cu-SDS/PGE electrodes had hierarchical dendritic structures of copper NPs, thereby increasing the surface area and electrochemical catalytic activity in comparison with Cu/PGE electrodes. The Cu-SDS/PGE electrode showed excellent catalytic activity in reducing hydrogen peroxide, resulting in the sensitive and selective detection of hydrogen peroxide. The electrode exhibited a good sensitivity of 21.42 µA/µM/cm^2^, a lower limit of detection 0.35, and a response time of less than 2 s over a wide range spanning 1 µM to 1 mM of hydrogen peroxide concentrations. The electrodes were also highly selective for H_2_O_2_ with minimal interference from other analytes even at concentrations higher than that of H_2_O_2_. The approach offers the benefit of electrode preparation in just 5 min, followed by analysis in 10 min, and enables for the quantitative determination of hydrogen peroxide within 30 min. This can be achieved utilizing a newly prepared, cost-effective electrode without the need for complex procedures.

## 1. Introduction

Hydrogen peroxide (H_2_O_2_) is essential for oxidative stress mechanisms, immunological responses, and cellular communication pathways. Detecting H_2_O_2_ is crucial for understanding biological processes and diagnosing disorders such as cancer, diabetes, and neurological issues [1,2]. Finding reliable means to detect H_2_O_2_ became a major scientific focus due to its importance. H_2_O_2_ is conventionally detected by spectrophotometry, fluorometry, and chemiluminescence [3,4,5]. While these methodologies exhibit high sensitivity and selectivity, their applications are often limited by expensive instrumentation, complex sample preparations and skilled labor. These limitations highlight the need for more accessible, cost-effective, and straightforward detection approaches [6].

On the other hand, electrochemical techniques such as enzymatic [7] and non-enzymatic [8] have emerged as viable substitutes for fast H_2_O_2_ detection with their intrinsic advantages (rapid response times and high sensitivity and selectivity) [9]. The key drawbacks of both enzymatic and most non-enzymatic sensors are multistep, complex sensor design involving tedious cleaning processes, and immobilization of sensing material (e.g., enzymes) on the electrode surface that requires extensive processing time and limited cycle life. Better response time at a lower cost was proposed for non-enzymatic sensors made with nanocatalyst loading [10].

The use of noble metal nanostructures, such as gold, platinum, and palladium, has increased over the past few years, considering their extensive application in catalysis and electrocatalysis [11,12]. Although these metals exhibit great performance, the high cost of these elements hampers their widespread application. By contrast, copper-based nanostructures have gained more prevalence; they are relatively cheap, stable, and good for catalytic applications. Copper oxide is a p-type semiconducting material with a band gap of 1.2 eV and shows excellent mechanical, thermal and electrical properties and can be used in a wide range of applications, including sensors, catalysis and other field emission devices [13,14].

The sensitivity and efficacy of a sensor in terms of the catalytic materials used are largely dependent on the active surface area. In the literature, many methods are used to increase the surface area such as chemical and physical hydrothermal and electrochemical processes. Among these, electrochemical methods stand out due to the requirement of sophisticated conditions such as high temperatures and vacuum [15]. Surfactants serve as ideal templates for electrochemical deposition due to their polymorphic structures and long-range periodicities, typically ranging from 2 to 15 nm [16,17]. The use of these capping agents decreases the surface tension in the precursor solution, inducing nucleation and forming a multitude of nanoparticle phases. This templated deposition method has been used widely for various metals, like depositing platinum on the use of lyotropic liquid crystal phases, palladium with nonionic surfactants, and nickel deposition on liquid crystalline phases. This approach enhances surface areas with uniform pore sizes and improves electrocatalytic/analytic properties [11,18].

Additionally, electrochemical methods offer advantages including the ability to tailor morphology by using different templates as electrolytes or by adjusting the voltage, current, or coulombic charge during deposition. Furthermore, electrodeposition makes it possible for nanoparticles to develop on a variety of substrates, such as conducting polymers, semiconductors (like ITO), conductors (including copper, platinum electrodes, and boron-doped diamond), and polyaniline-covered PET. The shape of the deposited copper nanostructures is greatly influenced by the substrate selection. Notably, pencil graphite electrode (PGE) is a preferred electrode material because of its low cost, mechanical stiffness, strong electrochemical activity, and commercial availability, which makes it ideal for disposable applications [19,20]. Moreover, PGE substrates can be easily modified, making them useful for immunoassays and pharmaceutical analysis. In this work, we opted to electrodeposit copper nanostructures solely using the anionic surfactant sodium dodecyl sulfate (SDS). This choice is supported by strong data from current studies, showing that SDS (anionic), as opposed to cationic and non-ionic surfactants, greatly increases the electrochemical surface area of the deposited copper nanostructures [20]. High hydrophilic-lipophilic balance (HLB) of SDS (40) also acts as a driving force in fine tuning the morphology of the electrodeposited material.

Herein, we demonstrate a simple pencil graphite electrode template electrodeposited with copper–copper oxide (Cu-Cu_2_O) nanostructures. Hydrogen peroxide was detected electrochemically using such manufactured electrodes. This quick five-minute preparation and analysis procedure offers one of the quickest ways to analyze hydrogen peroxide with newly fabricated electrodes.

## 2. Results and Discussion

### 2.1. Physical Characterization

**Figure 1:**
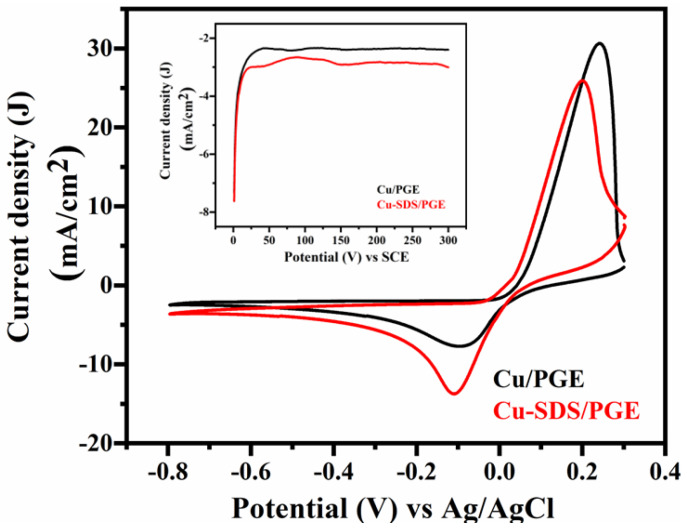
Cyclic voltammograms of pencil graphite electrode (PGE) in the presence (Cu-SDS/PGE) and absence (Cu/PGE) of SDS in a copper electroplating bath at 50 mV/s scan rate. The inset shows the amperometric response obtained during the electrodeposition of copper at constant applied potential of −0.7 V vs. SCE.

Figure 1 represents the cyclic voltammograms of a pencil graphite electrode (PGE) in a solution containing 15 mM CuCl_2_ solution and 0.1 M KCl and the influence of anionic surfactant (SDS) over copper redox peaks. It was observed that the copper cathodic peak appears at close to −0.08 V vs. Ag/AgCl in the absence of surfactant. However, the reduction peak was shifted to a more negative potential (−0.13 V) in the presence of SDS. The shift in the redox peaks can be attributed to the interactions of the surfactant with electrodes and/or copper ions. In order to minimize the influence of reduction potential on the shape of the deposited material, the process of electrodeposition was carried out at a considerably higher negative potential than the point at which the cathodic peak occurs. Copper nanostructures were achieved using the electrodeposition method at a higher negative potential by constantly applying −0.7 V vs. SCE for five minutes. A model chronoamperogram is shown in Figure 1 (inset). The potential for electrodeposition and time was optimized with a detailed discussion already presented elsewhere [20]. In brief, the copper deposition was performed at different deposition potentials viz. −0.5 V, −0.7 V, and −0.9 V vs. SCE. The rationale behind choosing this potential lies in its ability to balance between effective reduction of Cu^2+^ ions and minimizing HER activity, thereby enhancing nanostructure quality. When SDS was used as a soft template, coulombic interactions can take place between the anionic head group of the surfactant and the copper cation, significantly altering the diffusion coefficient of Cu^2+^ ions in the electrolyte [19].

**Figure 2:**
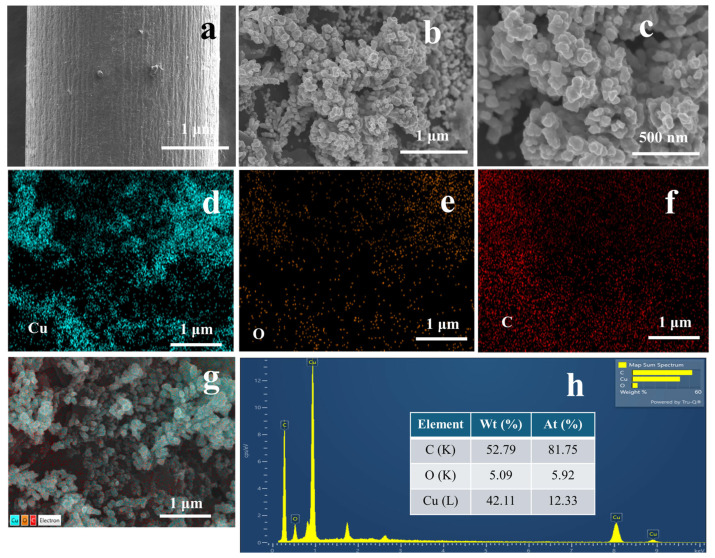
FE-SEM images of bare PGE (**a**), Cu/PGE (**b**,**c**) at different magnifications. Elemental mapping of Cu, O, and C on Cu/PGE (**d**–**f**) with corresponding SEM image (**g**). EDX spectrum showing the elemental composition of Cu/PGE (**h**).

The scanning electron microscope (SEM) images clearly illustrate distinct variations in the morphology of the copper nanostructures electrodeposited on PGE with and without presence of SDS. Bare PGE exhibits planar surface without any significant surface features, as shown in Figure 2a. Upon copper deposition (in the absence of SDS), the surface morphology changes drastically. Partial dendritic structures were observed, suggesting that the coverage is not uniform, and that the porosity is relatively moderate as shown in Figure 2b,c. This morphology is due to the uncontrollable nucleation and growth of copper in the absence of surfactant, causing random spot deposition.

Figure 2d–g display the energy dispersive X-ray (EDX) examination of Cu-PGE, showing the distribution of copper, oxygen, and carbon on the electrode surface along with corresponding SEM images. The oxygen (O) and carbon (C) maps offer supplementary information; the presence of oxygen is ascribed to the creation of oxides during or after deposition, while the carbon signal corresponds to the underlying graphite substrate. The EDX spectrum (Figure 2h) provides quantitative evidence that supports these conclusions. The spectrum has prominent peaks corresponding to carbon and copper, as well as a minor peak corresponding to oxygen. The weight % data confirms that copper makes up a significant proportion of the modified electrode, indicating successful deposition. The presence of oxygen, albeit in lesser quantities, indicates the possibility of copper oxidation or the presence of residual contaminants from the electroplating solution.

**Figure 3:**
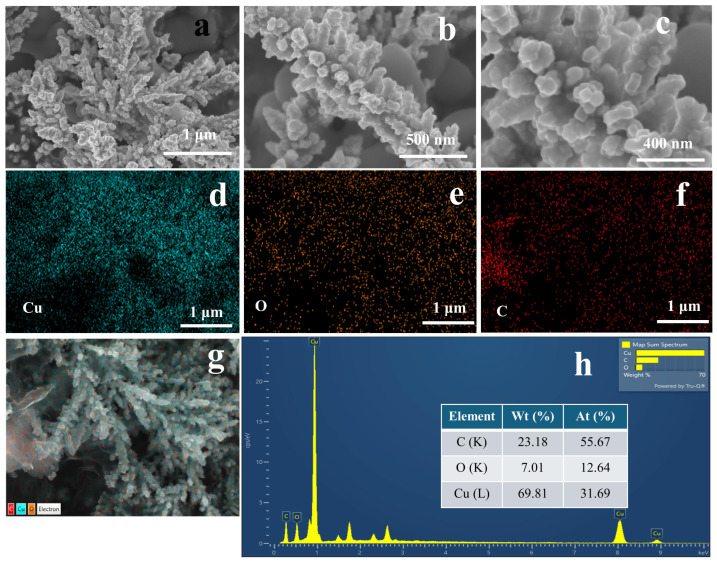
FE-SEM images of Cu-SDS/PGE (**a**–**c**) at different magnifications. Elemental mapping of Cu, O, and C, respectively, on Cu-SDS/PGE (**d**–**f**) with corresponding SEM image (**g**). EDX spectrum showing the elemental composition of the Cu-SDS/PGE (**h**).

The SEM images in Figure 3 demonstrate the impact of adding sodium dodecyl sulfate (SDS) to the electrolyte solution when depositing copper onto the PGE. The SEM images of Figure 3a–c show distinct dendritic morphologies that significantly increased the active surface area and porosity. The dendrites exhibit clear and consistent distribution over the electrode surface, in contrast to that of Cu/PGE. The elemental mapping as depicted in Figure 3d–g provides evidence of copper distribution throughout the electrode surface, resembling the Cu/PGE sample. Nevertheless, the Cu-SDS/PGE exhibits a little more prominent oxygen content, as evidenced by the O mapping (Figure 3e) and EDX spectrum (Figure 3h). This suggests the development of a thin layer of oxide, either caused by the interaction between SDS and the copper during the deposition process and/or subsequent oxidation.

The observed variation in shape can be ascribed to the existence of the anionic surfactant SDS, which has a critical influence on controlling the copper-deposition procedure. The EDX mapping further supports these observations. For Cu/PGE, the copper content is 42.11% by weight and 12.33% by atomic percentage, whereas Cu-SDS/PGE demonstrates a higher copper content of 69.81% by weight and 31.69% by atomic percentage. The elevated copper loading observed in Cu-SDS/PGE suggests that the presence of SDS greatly improves the efficiency of deposition, possibly by influencing the diffusion of copper ions and the kinetics of nucleation.

In the absence of SDS, the copper ions in the electrolyte solution directly reach the electrode surface through a classical diffusion-controlled mechanism, resulting in irregular growth of copper nanostructures. The inclusion of SDS in the electrolyte can significantly influence the deposition process by altering the surface interactions and deposition kinetics. The presence of SDS above its CMC enhances the nucleation rate by micelle-mediated mass transport, while simultaneously inhibiting the random aggregation of copper atoms. These micellar interactions can localize copper ions in the immediate vicinity of the electrode surface, which in turn affects the diffusion coefficient of Cu^2+^ ions. This change in local ion concentrations can alter the nucleation overpotential and growth kinetics of the copper-deposition process. The micelles serve as soft templates, providing a scaffold that guides the development of copper nanostructures in a controlled manner. Additionally, surfactant molecules can adsorb onto the electrode surface, reducing the surface energy and facilitating the growth of high surface area nanostructures. The nucleation overpotential decreases as a result of the reduction in the surface tension which also can enhance the growth orientations. Therefore, SDS not only acts as a mediator of ion transport but also plays a crucial role in shaping the morphology [21,22].

**Figure 4:**
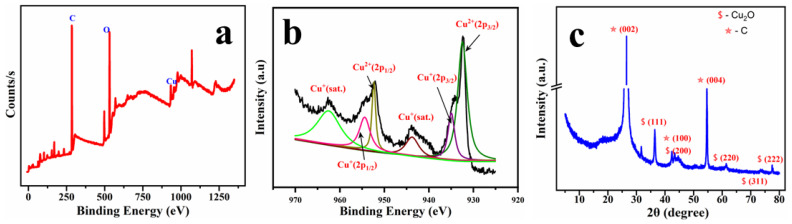
XPS survey spectra (**a**) and elemental spectra of Cu (**b**) and the XRD patterns (**c**) of the electrodeposited Cu-SDS/PGE (JCPDS; Cu-01-078-2076 and C-00-041-1487).

Figure 4a shows the XPS survey spectra of the designed electrocatalyst (Cu-SDS/PGE) and depicts three elements: C(1s), Cu(2p), and O(1s). The presence of carbon could be attributed to the underlying graphite electrode and the oxygen is due to the formation of oxides following the electrodeposition. Furthermore, the corresponding high-resolution spectra of Cu(2p) peaks are presented in Figure 4b along with their deconvolution. In the Cu 2p spectra, the peaks observed at 932.36 eV and 952.11 eV are attributed to the Cu 2p3/2 and Cu 2p1/2 orbitals of metallic copper. The disparities in binding energy (Δ = 19.75 eV) between the Cu 2p3/2 and Cu 2p1/2 orbitals align well with the previously reported values for metallic copper/zerovalent Cu [23,24]. Shakeup satellites were observed in the Cu 2p spectra of Cu np at binding energies of 962.70 eV and 943.65 eV. This is a distinctive feature of the substance containing Cu^2+^ ions in the form of CuO. Thus, we confirm the presence of both metallic copper (Cu) and copper oxide (CuO) on the surface of Cu-SDS/PGE. The XRD pattern verifies the effective electrodeposition of Cu_2_O on the pencil graphite electrode as shown in Figure 4c. This is evident from the distinct peaks observed at 2θ ≈ 29.6°, 36.4°, 42.3°, 61.4°, 73.6°, and 77.3°, which correspond to the Cu_2_O (110), (111), (200), (220), (311), and (222) planes, respectively [25,26]. The presence of the underlying graphite structure is confirmed by the distinct graphite peak at 26.5° (002).

The electrochemical surface area was estimated by means of double-layer capacitance measurements through cyclic voltammograms at different scan rates (10 mV/s to 100 mV/s) in 0.5 M HClO_4_ solution. The capacitance values were determined by analyzing the slope of current density-scan rate plots. From these values, the roughness factor (RF) can be correlated with specific capacitance value (C*_ref_*) of 29 μF·cm^−2^ for polycrystalline copper samples [27]. The estimated RF values for Cu/PGE and Cu-SDS/PGE are 18.33 and 21.28, respectively.

### 2.2. Electrochemical Studies

**Figure 5:**
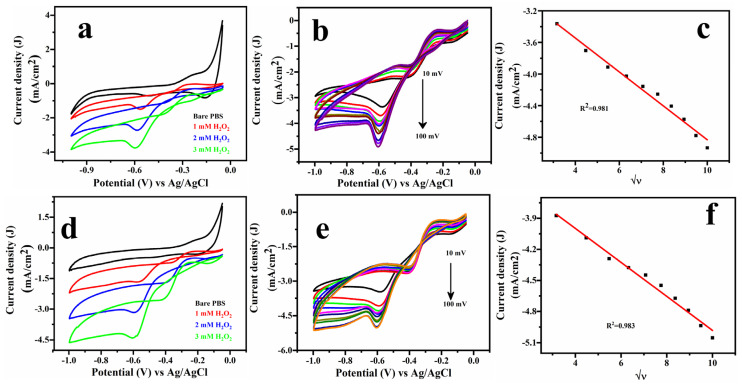
Cyclic voltammogram (CV) for nitrate reduction using Cu/PGE (**a**–**c**) and Cu-SDS/PGE (**d**–**f**). (**a**,**d**) CV responses with increasing nitrate concentrations; (**b**,**c**,**e**,**f**) the relationship between peak current and scan rate.

The electrochemical efficiency of Cu/PGE and Cu-SDS/PGE electrodes in detecting hydrogen peroxide (H_2_O_2_) was assessed using cyclic voltammetry (CV) in phosphate buffer saline (0.1 M PBS, 7.4 pH) as displayed in Figure 5. Figure 5a,d display the CV curves for the Cu/PGE and Cu-SDS/PGE electrodes in pure PBS and with increasing concentrations of H_2_O_2_ (1–3 mM). A significant cathodic peak was observed, corresponding to hydrogen peroxide reduction at around −0.6 V vs. Ag/AgCl (Sat. KCl) for both the electrodes. However, the Cu-SDS/PGE exhibited enhanced current density, indicating improved sensitivity due to SDS modification. The scan rate-dependence analysis demonstrates a linear correlation between peak current density and the square root of the scan rate for both electrodes, demonstrating that the electrochemical processes are governed by diffusion for both electrodes, as demonstrated in Figure 5b–f. The SDS modification enhances the surface characteristics of the electrode, leading to improved electrochemical response while maintaining the diffusion-controlled nature of the process [28].

**Figure 6:**
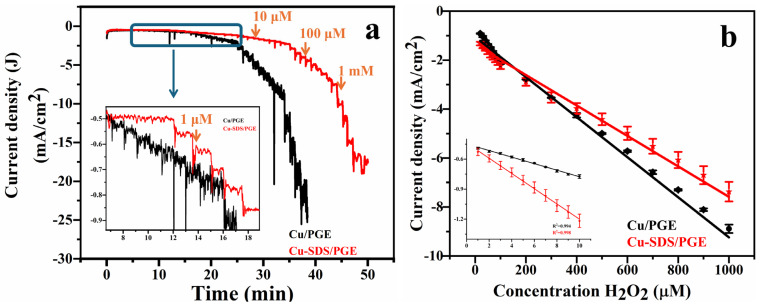
Amperometric i-t response curves of Cu/PGE and Cu-SDS/PGE at applied potential of −0.6 V vs. Ag/AgCl with successive addition of H_2_O_2_ from 1 µM to few mM concentration in 0.1 M PBS and 0.1 M KCl (**a**) and their corresponding concentration vs. current response of H_2_O_2_ calibration graphs (**b**). Inset of (**a**) represents the amperometric response at low concentrations (1 to 10 µM) and (**b**) inset represents the calibration graph at lower concentrations (1 to 10 µM).

In order to obtain the quantitative relationship between concentration of hydrogen peroxide and the faradaic current, chronoamperometric studies were performed with successive addition of analyte at a constant applied potential of −0.6 V vs. Ag/AgCl (Sat. KCl). Figure 6a displays the amperometric response of the Cu/PGE (black curve) and Cu-SDS/PGE (red curve) electrodes to varying concentrations of H_2_O_2_, ranging from 1 µM to 5 mM. The highlighted section, depicted in the inset of Figure 6a, specifically focuses on the response at lower concentrations of H_2_O_2_. The response indicates that the two electrodes are sensitive towards H_2_O_2_, with the Cu-SDS/PGE electrode exhibiting a more pronounced current density compared to Cu/PGE. This amplified response of Cu-SDS/PGE can be credited to the existence of SDS, which likely improves the electrode’s surface qualities and interaction with H_2_O_2_. The enhanced reaction of the Cu-SDS/PGE electrode demonstrates the impact of surface modifications on sensor performance.

Figure 6b elucidates the calibration graphs derived from the triplicate analysis of the electrodes. Both Cu/PGE and Cu-SDS/PGE exhibit two distinct linear variations in their calibration curves. Instead of a single linear range, the electrodes exhibit multiple linear ranges from 1 µM to 10 µM, from 20 µM to 100 µM and 200 µM to 5 mM for both the electrodes. The reason for exhibiting multiple linear ranges could be ascribed to mixed controlled reaction rate and the kinetics of the electrode interaction with the hydrogen peroxide. Moreover, different oxidation states of copper (0, +1, +2) could also affect the linear ranges [29,30]. The inset in Figure 6b offers a close-up examination of the calibration graph at lower concentrations, validating the electrodes’ exceptional sensitivity and linearity within the 1 to 10 µM range. The Cu-SDS/PGE electrode exhibits superior sensitivity and improved linearity (R^2^ = 0.998) at lower concentration levels, in contrast to the Cu/PGE electrode (R^2^ = 0.994). This suggests that the addition of SDS greatly improves the performance of the electrode in detecting low levels of H_2_O_2_. The enhanced performance can be ascribed to the surfactant characteristics of SDS, which presumably augment the effective surface area and accelerate the electron transfer kinetics of the Cu-SDS/PGE electrode. Table 1 contains comprehensive data on the sensor parameters derived from these figures. Based on the information presented in Table 1, it is evident that Cu-SDS/PGE offers superior sensitivity of 21.42 µA/µM/cm^2^ and lower limit of detection (LOD defined as 3xSTD (standard deviation) of baseline/sensitivity) as 0.35 µM ± 0.04 µM. In comparison, Cu/PGE exhibited sensitivity of 14.82 µA/µM/cm^2^ and LOD of 0.92 µM ± 0.07 µM.

Several materials used for the electrochemical detection of hydrogen peroxide (H_2_O_2_) are compared in Table 2 solely on potential, linear range, limit of detection (LOD), and sensitivity. In our study, we present two new electrodes in our study, Cu/PGE and Cu-SDS/PGE, with a potential of −0.6 V, a linear range of 1–1000 µM, and respective LODs of 0.92 µM and 0.35 µM. These results are competitive compared with other methods, such as a Cu-BTC/GN/GCE with LOD of 2 µM, or the Ag Nps-PANINRs with LOD of just 0.13 µM, highlighting that our electrodes offer a balance between detection range and sensitivity. More importantly, our method is easy to conduct and analyze; it involves simple electrochemical deposition of copper nanostructures, which is completed within just 5 min, as opposed to the long preparation of Cu-BTC/MPC/GCE or MIL-53-CrIIIAs/GCE. Furthermore, those complex materials both require intricate synthetic processes and coating procedures. Also, the potential for onsite analysis with our Cu/PGE and Cu-SDS/PGE electrodes is distinctly greater due to their simplicity and rapid turnaround. This makes them particularly suitable for real-world applications where quick and efficient detection is mandatory.

**Figure 7:**
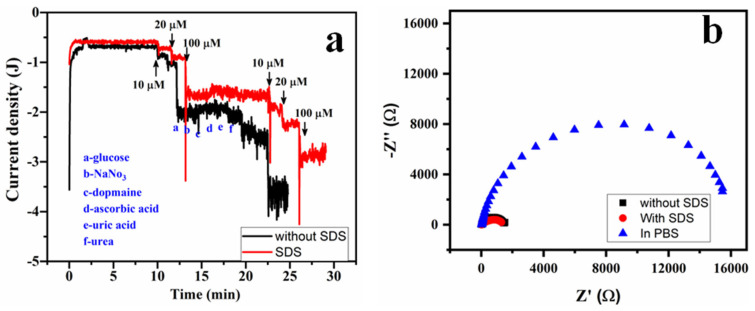
Interference studies of Cu/PGE and Cu-SDS/PGE electrodes for H_2_O_2_ detection in 0.1 M PBS with addition of common interfering agents (**a**) and electrochemical impedance spectra of fabricated electrodes in the presence and absence of H_2_O_2_ (**b**).

In order to determine the selectivity of the copper-modified pencil graphite electrodes towards H_2_O_2_, interference measurements were conducted during amperometric i-*t* tests. In the interference studies, a fixed potential of −0.6 V vs. Ag/AgCl reference electrode was maintained while gradually introducing H_2_O_2_. During the amperometric examination of hydrogen peroxide, many substances that commonly interfere with biological systems, such as glucose, dopamine, ascorbic acid, uric acid, urea, as well as common ionic species including nitrates, were intentionally added. The data depicted in Figure 7a demonstrate a favorable reaction at lower concentrations of 10 µM and 100 µM H_2_O_2_. However, the response to interfering agents at significantly higher concentrations (10 times more than the concentration of the analyte; 1 mM) was nearly insignificant. In addition, it was noted that even when there were high levels of interfering agents present, the performance of both the copper electrodes was not affected. This demonstrates the high level of specificity that copper electrodes have towards hydrogen peroxide. Figure 7b represents the impedimetric measurements (EIS) of Cu/PGE and Cu-SDS/PGE electrode with the presence and absence of analyte. The Nyquist plots exhibited one semicircle indicating single electron-transfer reactions and the data are fitted using modified Randles circuit R(QR). Charge-transfer-resistance (R_ct_) values were extracted to be 16.64 kΩ for the electrode in PBS. However, the R_ct_ values for Cu/PGE and Cu-SDS/PGE are 1.5 kΩ and 1.3 kΩ, respectively, suggesting that the reaction is more facile in Cu-SDS/PGE with the addition of analyte.

To evaluate the practical applications of the proposed sensor, hydrogen peroxide determination was performed in milk samples. The milk samples were filtered using a 0.22-micron filter to remove any larger particles that could potentially interfere with the electrochemical measurements and the pH was adjusted to 7.4. Known concentrations of hydrogen peroxide (5, 10 and 15 µM) were spiked into the electrolyte and the recovery of the H_2_O_2_ concentrations was measured using chronoamperometry. The recovery of H_2_O_2_ concentration was then determined using chronoamperometry without any special pretreatment of the samples besides diluting them tenfold with 0.1 M PBS at pH 7.4. Since Cu-SDS/PGE showed better performance, the same was used for this experiment. Recovery studies showed results ranging from 102% to 109% with minimal error rates below 3%, indicating the suitability of the copper-based sensor for analyzing H_2_O_2_ in biological samples as displayed in Table 3. Recoveries exceeding 100% may be due to trace amounts of H_2_O_2_ already present in the samples, as well as matrix effects in the complex media.

## 3. Materials and Methods

### 3.1. Chemicals

Copper chloride CuCl_2_, sulphuric acid (H_2_SO_4_), sodium dodecyl sulphate (SDS), potassium chloride (KCl), potassium dihydrogen phosphate (KH_2_PO_4_), dipotassium phosphate (K_2_HPO_4_), glucose (C_6_H_12_O_6_), sodium nitrate (NaNO_3_), urea (NH_2_CONH_2_), ascorbic acid (C_6_H_8_O_6_), uric acid (C_5_H_4_N_4_NO_3_), and dopamine (C_8_H_11_NO_2_) were purchased from Sigma Aldrich (St. Louis, MO, USA) and were used without any purification. Hydrogen peroxide (H_2_O_2_) was procured from Junsei chemicals (Tokyo, Japan). Deionized water was used to prepare aqueous solutions unless mentioned. Polymerized pencil graphite (PGE, Faber Castell, Stein, Germany, 0.7 mm HB) was brought from a local stationary shop and used as substrate for catalyst modification. All chemicals used in this study were of analytical grade with high purity and were used as such without any modification. All aqueous solutions were made using HPLC water.

### 3.2. Experimental Methods

All electrochemical experiments were performed at room temperature using a three-electrode configuration with an electrochemical workstation (Zive Sp1). Either aqueous Ag/AgCl (satd. KCl) electrode and/or saturated calomel electrode (SCE, satd. KCl) were used as the reference electrodes and platinum foil as the counter electrode. A pencil graphite electrode (PGE) with a 1 cm^2^ exposed area was employed as the working electrode. The electrolyte bath for copper electrodeposition includes 0.1 M KCl as a supporting electrolyte, 10 mM of SDS (surfactant), and 15 mM of copper chloride as precursor. A constant potential of −0.7 V vs. SCE was applied to deposit copper nanostructures over a period of five minutes. After being removed from the solution, the copper-modified pencil graphite (Cu-SDS/PGE) was air-dried and cleaned with deionized water before being utilized for further studies. A control sample (Cu/PGE) was also prepared using similar conditions without addition of SDS.

The surface area and roughness of the copper nanostructures were determined by employing the double layer capacitance method through cyclic voltammetry at different scan rates (in 0.5 M perchloric acid solution). The charge-transfer-resistance values for the copper modified pencil electrodes (PGEs) during the reduction of hydrogen peroxide were determined using the electrochemical impedance spectroscopy (EIS) method. The measurements were conducted at a potential of −0.6 V vs. Ag/AgCl in a solution comprising 1 mM hydrogen peroxide in 0.1 M phosphate-buffered saline (PBS) and 0.1 M potassium chloride (KCl) as the supporting electrolyte. The EIS data were analyzed using ZSimpWin software (3.20) and fitted with a modified Randles circuit R(QR) and R(QR(W)).

Sensing studies were performed using modified PGE as working electrode in 15 mL of PBS (7.4 pH). Chronoamperometry was employed for the detection of hydrogen peroxide at an applied potential of −0.6 V vs. Ag/AgCl. Each experiment was conducted in triplicate in order to confirm the reproducibility of the sensor performance. Interference analyses were performed using the same conditions mentioned above.

### 3.3. Material Characterization

In order to determine the details of the fabricated catalyst, X-ray diffraction (XRD), X-ray photoelectron spectroscopy (XPS) and field emission scanning electron microscope with energy dispersive X-ray analysis (SEM-EDX) were used. XRD data measurements were obtained using D8 focus, Bruker, Germany, with Cu Kα source radiation of λ = 1.54060 Å. The data were taken in the 2θ range of 5° to 80° with a step of 0.05° to ascertain the crystalline phase of the material. Surface morphology and elemental composition analysis were obtained by SEM using FE-SEM, Hitachi-SU8600 microscope at the Smart Materials Research Center for IOT at Gachon University (Seongnam-si, Republic of Korea). Experimentation was conducted with an accelerating voltage of 0.5 to 30 kV; a microscope is fitted with Tungsten source and with EDX detector area of 30 mm^2^ in vacuum. XPS measurement was conducted with Thermo scientific K-alpha instrument to provide detailed information regarding catalyst surface elements and their electronic configuration. The chemical states on the sample surface were examined using XPS (XPS, Ulvac-PHI 5000, Kanagawa, Japan).

## 4. Conclusions

This paper presents a facile approach to preparing copper nanostructures using electrodeposition with a surfactant template. Notably, the addition of an anionic surfactant, SDS, significantly impacted morphology and electrochemical catalytic activity of the copper deposits. Examination with FE-SEM and EDX revealed the Cu-SDS/PGE electrode possessed distinct dendritic configurations with enriched copper contents compared to the SDS-free counterpart, Cu/PGE electrode. This led to an increase in surface area and porosity, hence enhancing its overall performance. The Cu-SDS/PGE electrode exhibited exceptional sensitivity and selectivity in detecting H_2_O_2_. This facile preparative approach, coupled with efficient electroanalysis of hydrogen peroxide, highlights one of the quickest and most effective techniques for hydrogen peroxide analysis using freshly prepared electrodes, emphasizing its potential for significant applications in sensitive and selective chemical sensing.

## Figures and Tables

**Table 1:** Comparison of sensor parameters of modified PGE electrodes for hydrogen peroxide analysis.

Electrode	Linear Range (µM)	Sensitivity (µA/mM/cm^2^)	LOD (µM)
Cu/PGE	1–10	14.82	0.92 ± 0.07
	20–100	6.8	
Cu-SDS/PGE	1–10	21.42	0.35 ± 0.04
	20–100	5.27	

**Table 2:** Comparison of the sensing performance of various reported nanomaterials towards electrochemical detection of hydrogen peroxide.

Electrode	Potential (V)	Linear Range (µM)	LOD (µM)	Sensitivity (µA/mM/cm^2^)	Ref.
Cu-BTC/GN/GCE	−0.35	10–11,180	2	57.73	[31]
Cu-BTC/MPC/GCE	−0.23	10–11,600	3.2	2.97	[32]
Ag Y/CPE	−0.65	10–5000	1.4	650.7	[33]
Ag Nps/Nafion	−0.35	100–8000	0.48	-	[34]
Ag Nps-PANINRs	−0.35	100–1000	0.13	-	[35]
MIL-53-CrIIIAs/GCE	DPV	25–500	3.52	11.9	[36]
NiFe-LDH/Ni foam	0.4	0.5–0.84	0.5	1704	[37]
Cu-TCPP MOF	−0.7	0.1–600	0.13	-	[38]
Cu/PGE	−0.6	1–1000	0.92	14.82	This work
Cu-SDS/PGE	−0.6	1–1000	0.35	21.42	This work

**Table 3:** Recovery data for real sample analysis using Cu-SDS/PGE electrode in milk samples, where known amount of H_2_O_2_ was spiked into the sample.

Quantity Spiked (µM)	Detected (µM)	Recovery (%)	RSD (%)
5	5.36	109.6	2.52
10	10.62	106.2	1.55
15	15.33	102.2	1.23

## Data Availability

The original contributions presented in the study are included in the article, further inquiries can be directed to the corresponding author.
